# Noninvasive Analysis of Tree Stems by Electrical Resistivity Tomography: Unraveling the Effects of Temperature, Water Status, and Electrode Installation

**DOI:** 10.3389/fpls.2019.01455

**Published:** 2019-11-13

**Authors:** Andrea Ganthaler, Julia Sailer, Andreas Bär, Adriano Losso, Stefan Mayr

**Affiliations:** Department of Botany, University of Innsbruck, Innsbruck, Austria

**Keywords:** angiosperms, conifers, imaging, nondestructive, ring electrode array, tree assessment, water status

## Abstract

The increasing demand for tree and forest health monitoring due to ongoing climate change requires new future-oriented and nondestructive measurement techniques. Electrical resistivity (ER) tomography represents a promising and innovative approach, as it allows insights into living trees based on ER levels and ER cross-sectional distribution patterns of stems. However, it is poorly understood how external factors, such as temperature, tree water status, and electrode installation affect ER tomograms. In this study, ER measurements were carried out on three angiosperms (*Betula pendula*, *Fagus sylvatica*, *Populus nigra*) and three conifers (*Larix decidua*, *Picea abies*, *Pinus cembra*) exposed to temperatures between −10 and 30°C and to continuous dehydration down to −6.3 MPa in a laboratory experiment. Additionally, effects of removal of peripheral tissues (periderm, phloem, cambium) and electrode installation were tested. Temperature changes above the freezing point did not affect ER distribution patterns but average ER levels, which increased exponentially and about 2.5-fold from 30 to 0°C in all species. In contrast, freezing of stems caused a pronounced raise of ER, especially in peripheral areas. With progressive tree dehydration, average ER increased in all species except in *B. pendula*, and measured resistivities in the peripheral stem areas of both angiosperms and conifers were clearly linearly related to the tree water status. Removal of the periderm resulted in a slight decrease of high ER peaks. Installation of electrodes for a short period of 32–72 h before conducting the tomography caused small distortions in tomograms. Distortions became serious after long-term installation for several months, while mean ER was only slightly affected. The present study confirms that ER tomography of tree stems is sensitive to temperature and water status. Results help to improve ER tomogram interpretation and suggest that ER analyses may be suitable to nondestructively determinate the hydraulic status of trees. They thus provide a solid basis for further technological developments to enable presymptomatic detection of physiological stress in standing trees.

## Introduction

The increasing demand for tree and forest health monitoring in terms of biotic and abiotic stresses (Allen et al., 2010; Jactel et al., 2012) calls for new sustainable and nondestructive measurement and monitoring techniques. New approaches are needed for early, potentially presymptomatic, diagnoses of reduced tree vitality and physiological stress responses. Electrical resistivity (ER) tomography represents a promising approach, as it offers detailed insights into trees *in vivo* and *in situ*, and it is nearly nondestructive (Daily et al., 2005). However, its application in plant sciences and especially tree physiology has not yet been sufficiently investigated and most studies focused on single species or even tree individuals (e.g. Bieker et al., 2010; Guyot et al., 2013; Martin and Günther, 2013; Goncz et al., 2018). A better knowledge of opportunities and limitations of the method would be a prerequisite to implement ER measurements for a wide spectrum of applications, ranging from tree assessment and determination of wood quality to the quantification of physiological processes in forests.

ER tomography was originally developed as a geophysical technique for subsurface investigations. Geological variations, such as fracture zones, soil lithology, presence of ground water and areas of increased salinity, can be detected by generating an electrical field and the subsequent measurement of the electrical conductivity (Revil et al., 2012). Methodological adaptations facilitated the introduction of ER techniques into plant and environmental sciences. ER measurements can, for example, nowadays be used to quantify and visualize root development, soil–root interactions, and plant water use (Weigand and Kemna, 2017; Vanella et al., 2018). Application to standing trees was enabled by further development and adaptation of the method by Tattar et al. (1972), Shigo and Shigo (1974), Just and Jacobs (1998), and Al Hagrey (2007). The latest measurement principle is based on a circular array of electrodes around the stem (multielectrode ring array), connected to the sapwood by small inserted nails. Step by step, an electrical field is induced by two electrodes and registered pairwise by the other inserted electrodes. The electrical conductivity, or its reciprocal the ER, is calculated for each measurement. Measured data are processed based on an inversion algorithm, which uses triangular meshes between the measurement points, to obtain the cross-sectional ER distribution (Günther et al., 2006; Martin and Günther, 2013). ER distribution patterns can then be visualized in a two-dimensional tomogram using a color scale.

Wood is a natural material with a highly complex anatomical structure and biochemical composition that can vary substantially between species but also between individual specimens. Due to this complexity, it is impossible to calculate theoretically ER of wood samples or to derive the effects of internal and external factors on the dielectric properties of wood on a theoretical basis (Torgovnikov, 1993). Nevertheless, electrical properties can be obtained by proper experimentation. Several studies showed that variation of ER in the tree stem is mainly caused by a heterogeneous distribution of moisture, electrolytes, and wood density in the cross section (Al Hagrey, 2007; Bieker and Rust, 2010b; Bieker et al., 2010; Guyot et al., 2013). Low ER thereby indicates higher wood moisture content, higher electrolyte content, and/or lower wood density. However, the impact of individual factors on the wood electrical properties can show broad variation, depending on the tree species (e.g. Bieker and Rust, 2010b; Guyot et al., 2013; Bär et al., 2019) and factors can superimpose or mask each other, complicating the analysis.

Since wood moisture, electrolyte content and cell structures are strongly altered by bacterial and fungal infections, wood decay, or cracks in the trunk, ER tomography has been successfully developed as a tool for the detection of microbial decay in both angiosperms (e.g. Nicolotti et al., 2003; Bieker et al., 2010; Brazee et al., 2011; Martin and Günther, 2013) and conifers (Larsson et al., 2004; Wunder et al., 2013; Humplík et al., 2016). The method can replace or complement invasive tools to assess internal wood decay in living trees, such as increment coring and subsequent testing by staining or use of a fractometer, and the application of resistance drilling (Rust, 1999). Like other tomographic methods (sonic, ultrasonic, and gamma ray computed tomography; Bieker et al., 2010), ER tomography is regarded as a nondestructive technology, because it requires solely the insertion of thin nails in the outer sapwood. As an additional benefit, ER measurements enable high resolution analysis and visualization of the resistivity distribution over entire cross sections in two- and three-dimensional formats. Besides the assessment of wood decay and cavities, ER tomography was also applied to differentiate sapwood from heartwood in several angiosperm and conifer species (Rust, 1999; Bieker and Rust, 2010a; Guyot et al., 2013; Wang et al., 2016). The transition zone from low ER in the periphery to high ER towards the center is assumed to indicate the sapwood–heartwood boundary, although the accuracy of the method is under debate (e.g. Pfautsch and Macfarlane, 2016). Furthermore, ER measurements were used to detect red heartwood formation in European beech (Goncz et al., 2018) and several other traits ranging from seasonal changes in cells to cold injury (see review of Gora and Yanoviak, 2015).

Notwithstanding the recent progress, many aspects of the application of ER tomography on tree stems are not yet fully understood. External conditions like temperature, tree water supply, bark characteristics, and electrode installation procedure are known to potentially influence ER measurements (Shigo and Shigo, 1974; Larsson et al., 2004; Guyot et al., 2013), but were never analyzed systematically and for a broad variety of species. Thus, it is difficult to differentiate effects of individual factors on the measured wood resistivity, to identify clear signals for abiotic and biotic stresses, and to implement the ER method in tree physiological analyses. ER measurements could help, e.g., to monitor the tree water status and to detect critical levels of drought stress in standing trees. In the present study, we analyzed the effects of temperature, plant water status, bark removal, and electrode installation on ER tomograms of tree stems. These aspects were addressed by applying ER measurements on six widespread and economically important European tree species, including three angiosperms (*Betula pendula*, *Fagus sylvatica*, *Populus nigra*) and three conifers (*Larix decidua*, *Picea abies*, *Pinus cembra*). Analysis of several species should enable to draw general conclusions for the functional groups (angiosperms versus conifers), but required to reduce the number of measurements per species. Moreover, we evaluated if the ER technology could be a powerful tool for nondestructive monitoring of tree water status.

We hypothesized that i) temperature dependence of ER measurements is negligible above 0°C, but considerable below the freezing point, and ii) dehydration of tree stems leads to characteristic changes in ER values and distribution patterns, enabling a reliable determination of the water status by analysis of ER tomograms. In this context, dehydration patterns were expected to be more complex in angiosperms than in conifers because their wood structure is more complex. In addition, we hypothesized that iii) removal of periderm, phloem, and cambium and the duration of electrode installation affect ER patterns. Analyses should highlight the high potential of ER analysis in plant physiology and provide a solid basis for the correct interpretation of tree tomograms.

## Materials and Methods

### Plant Material

Analyses were performed on three angiosperm species (*B. pendula* Roth, *F. sylvatica* L., *P. nigra* L.) and three conifers [*L. deciduas* Mill., *P. abies* (L.) H. Karst., *P. cembra* L.]. All species are native in Central Europe and were sampled in Tyrol, Austria on sites around Innsbruck (791 m, 47°27′N/11°37′E; all angiosperms and *L. decidua*) and in the Sellrain valley (1,760 m, 47°09′N/11°07′E; *P. abies* and *P. cembra*). Vital trees with no visible damages, straight stems, and circular trunks were selected for measurements, presuming that they are representative for the respective species. Trunk diameter at breast height varied between 42 and 70 mm, tree height between 2 and 4 m, and tree age between approximately 15–20 years (**Supplementary Tables S1–S4**). Entire trees were cut above ground, immediately wrapped in cling film, and enclosed in dark plastic bags to avoid dehydration during transport to the laboratory. Sampling was conducted between April and September during or after rainy periods to ensure saturation of stems. In the laboratory, trunks were recut under water for approximately 10–20 cm (angiosperms) and 5–10 cm (conifers) at the base. Subsequently, trees were saturated overnight by placing them in a bucket filled with water.

A part of the measurements (see below) was performed on only one tree per species. We are aware of the fact that more replicates would be desirable but available laboratory space (where up to 4 m high trees had to be dehydrated) and equipment (temperature test chamber) did not allow analysis of additional specimens. Accordingly, our study does not focus on species-specific characteristics but on general trends observed in angiosperms and conifers.

### Electrical Resistivity Measurements

A multichannel resistivity system (PiCUS TreeTronic, Argus Electronic GmbH, Germany) was used to perform ER measurement using a dipole–dipole configuration (Daily et al., 2005) at a low-frequency current of 8.3 Hz and at voltage levels between 2 and 8 V. The system was attached to the tree with stainless steel nails installed equally distributed around the trunk at constant height above ground (“multielectrode ring array”). In order to achieve the highest resolution possible we applied 24 electrodes, the maximum of the used ER measurement system. Nails were sterilized before measurement and inserted through bark and cambium to the outermost wood until a firm contact with the sapwood was established.

Electrical voltages were applied step by step to all installed electrodes and data of the electrical field were processed with the Picus Software (PiCUS TreeTronic Q73, Argus Electronics GmbH, Germany) according to inversion algorithms by Günther et al. (2006). The smoothness level (to regularize the solution) was set to 100 and the mesh fineness (to specify the number of triangles between two measurement points) was set to 4 (for details see also Günther et al., 2006). The cross-sectional distribution of ER was then calculated based on a circular tree geometry. For each triangle area within the tomogram, determined by the measurement lines between the installed electrodes, its position, size, and ER value was then exported and visualized by the use of a color scale ranging from blue (low ER) over green and yellow to red (high ER). Scale range was adjusted for each species between the lowest and highest ER detected at 20°C and full saturation, respectively.

For the calculation of ER profiles, transects of 6 mm width were defined through the cross sections and values of all triangle areas with their center therein were extracted. Orientation of profiles was adjusted to exclude reaction wood zones and ER profiles were related to the relative radial position between stem center and cambium (0% = stem center, ± 100% = youngest year ring) to account for variations in diameter between individual trees.

For the determination of the average ER of each cross section (ER_mean_; Ωm), the weighted electrical resistivity (ER_w_; Ωm) of all individual triangles was calculated as

$${\text{ER}}_{\text{w}}=(\text{ER}\;*\;\text{A})/{\text{A}}_{\text{mean}}$$

where A (cm²) is the individual triangle area and A_mean_ (cm²) the mean area of all triangles. ER_mean_ (weighted mean for the entire cross section) was then calculated as the mean of ER_w_ of all cross-sectional triangles. In addition, ER_center_ and ER_peripheral_ were calculated as the mean of ER_W_ of triangles within 0–10% and 90–95% relative radial position, respectively. These areas were defined in order to capture representative values for heart- and sapwood regions and to exclude local ER variations around electrodes.

### Experimental Design

Three different experiments were performed to analyze effects of i) temperature, ii) dehydration, and iii) electrode installation as well as bark removal on ER tomography measurements. All analyses were conducted in the laboratory on cut-off trees and under controlled conditions, as described in the following.

*(i) Temperature:* A trunk segment of 20 cm length was cut from the basis of one saturated tree per species and immediately wrapped in cling film to avoid dehydration. Electrodes for ER measurements were installed at midway with equal distance to both segment ends, which consequently corresponded to a stem height of about 20–30 cm above ground on the standing tree. The trunk segment was then placed in a temperature chamber (Binder GmbH, Germany) and was exposed successively to temperatures of 30, 20, 10, 0, and −10°C. At each temperature level, samples were acclimated for 2 h, before electrodes were connected to the computer system and, after an additional acclimation of 30 min, ER measurements were performed. Temperatures were monitored with type-T thermocouples, which were installed in the chamber (n = 1) and inserted into the trunk center (n = 2; about 2 cm above and below electrodes) and connected to a data logger (Campbell Scientific, Ltd, UK). In a final step, ER measurements were repeated for each trunk a second time at 20°C to exclude potential long-term changes of the wood (e.g. due to moisture changes) during the measurement procedure. The restricted length of trunk samples was expected not to cause significant measurement errors, because boundary effects are small at ratios of distance to measurement level and radius ≥ 5 (Weihs et al., 2013).

*(ii) Dehydration:* One saturated tree per species was unpacked, taken out of the water and mounted in a holder. Then each tree was continuously dehydrated at room temperature (20°C) and the water potential was measured regularly on three small terminal shoots per tree and time point with a Scholander Apparatus (Model 1000 Pressure Chamber, PMS Instrument Co., USA). In intervals of approximately 1 MPa, ER measurements were performed. For each ER measurement, a new electrode circle was installed a few centimeters below or above the old ones, to avoid aberrations due to long-term installation of electrodes (in the temperature experiment all measurements were performed on the same electrode circle because analyses were completed within 1 day).

*(iii) Bark removal and electrode installation:* To examine potential effects of the bark on ER measurements, a 25 cm long *F. sylvatica* trunk segment was equipped with nails and connected with electrodes to the measurement system like described in the temperature experiment. Following a first ER measurement, a layer of periderm and older phloem tissues was cautiously removed with a chisel within a band 1 cm above and below the nails and a second ER measurement was conducted. In the following, the periderm layer was removed first for 5 cm above and below the nail circle, then additionally the phloem and cambium was removed for 5 cm, and finally periderm, phloem, and cambium were removed for 10 cm above and below installed electrodes. ER measurements were conducted following each step. We choose *F. sylvatica* for this experiment as its bark consists of a periderm only and thus accurate installation of electrodes and removal of periderm parts was practicable.

In addition, effects of short- and long-term installation of electrodes on the ER tomogram were analyzed. Therefore, electrodes installed for the first ER measurement in the dehydration experiment remained on the tree (one individual per species). These electrodes were used to conduct a second measurement after 32–72 h, contemporaneously with a measurement on newly installed electrodes a few centimeters below or above. Besides that, electrodes remained installed on a living tree of *P. cembra* in the field for 1 year (September 2012–September 2013). After a period of about 10, 11, and 12 months, ER measurements were conducted and compared with tomograms gained by using newly installed electrodes.

### Statistics

Data were tested for Gaussian distribution (Kolmogorov–Smirnov test) and variance homogeneity (Levene test), and linear regressions of ER_mean_ (weighted mean of the entire cross section), ER_peripheral_ (weighted mean of peripheral areas), and ER_center_ (weighted mean of central areas; for details see chapter *ER Measurements*) versus the water potential were calculated with a simple linear regression model (conifer and angiosperm data were pooled) and fitted regression lines were visualized in the plots. Similarly, the regression of ER versus temperature was fitted with an exponential function (for details see the legend of **Supplementary Figure S1**). All tests were performed at a probability level of 5% using SPSS (version 24; SPSS, IL, USA).

## Results

### ER Tomography Patterns

**Figure 1:**
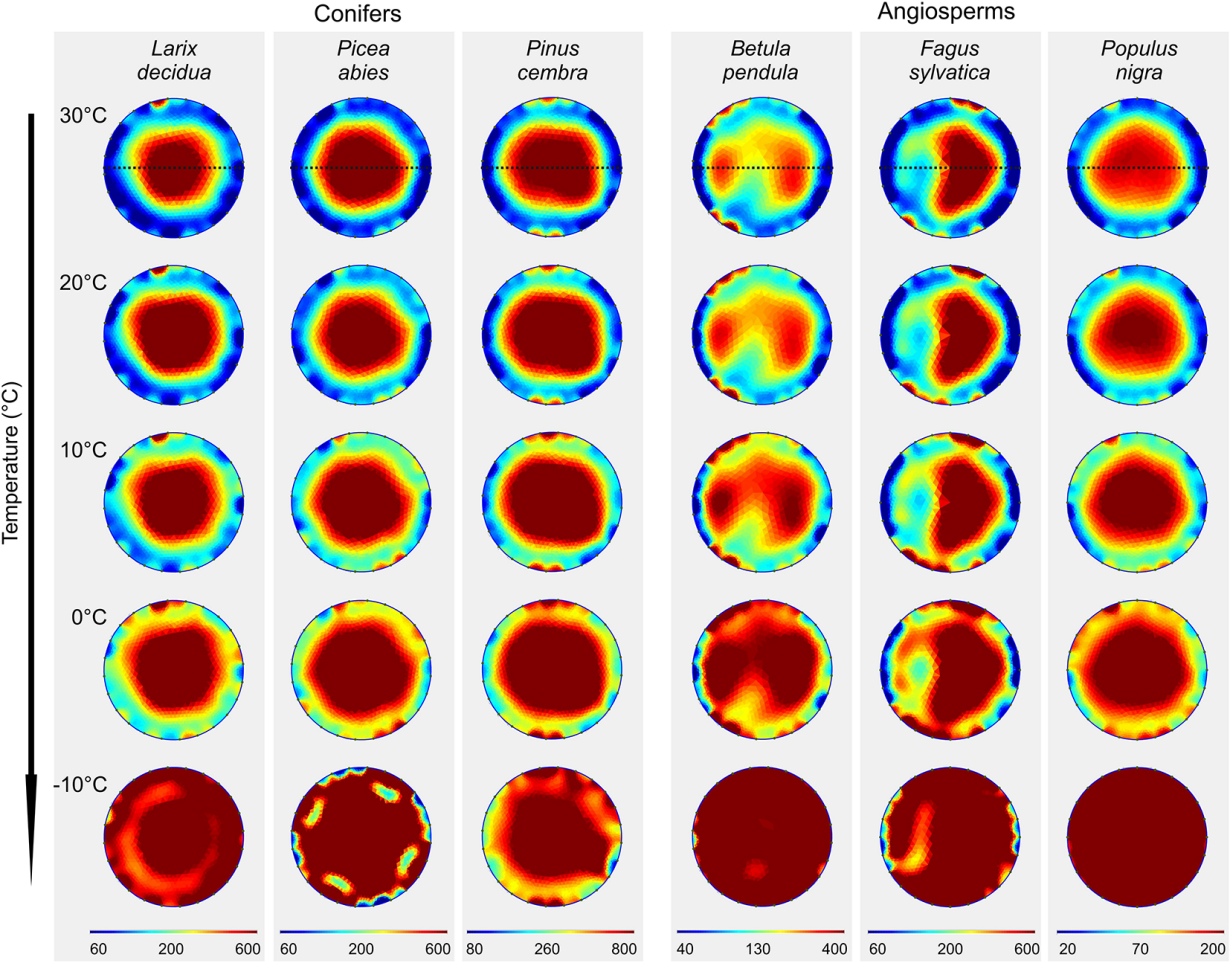
Electrical resistivity (ER) tomograms of the six analyzed tree species at different temperatures. Blue colors indicate areas of low, and red of high ER (Ωm; note the species-specific range of the scale). Dashed lines indicate the orientation of radial ER profiles shown in **Figure 2**.

**Figure 2:**
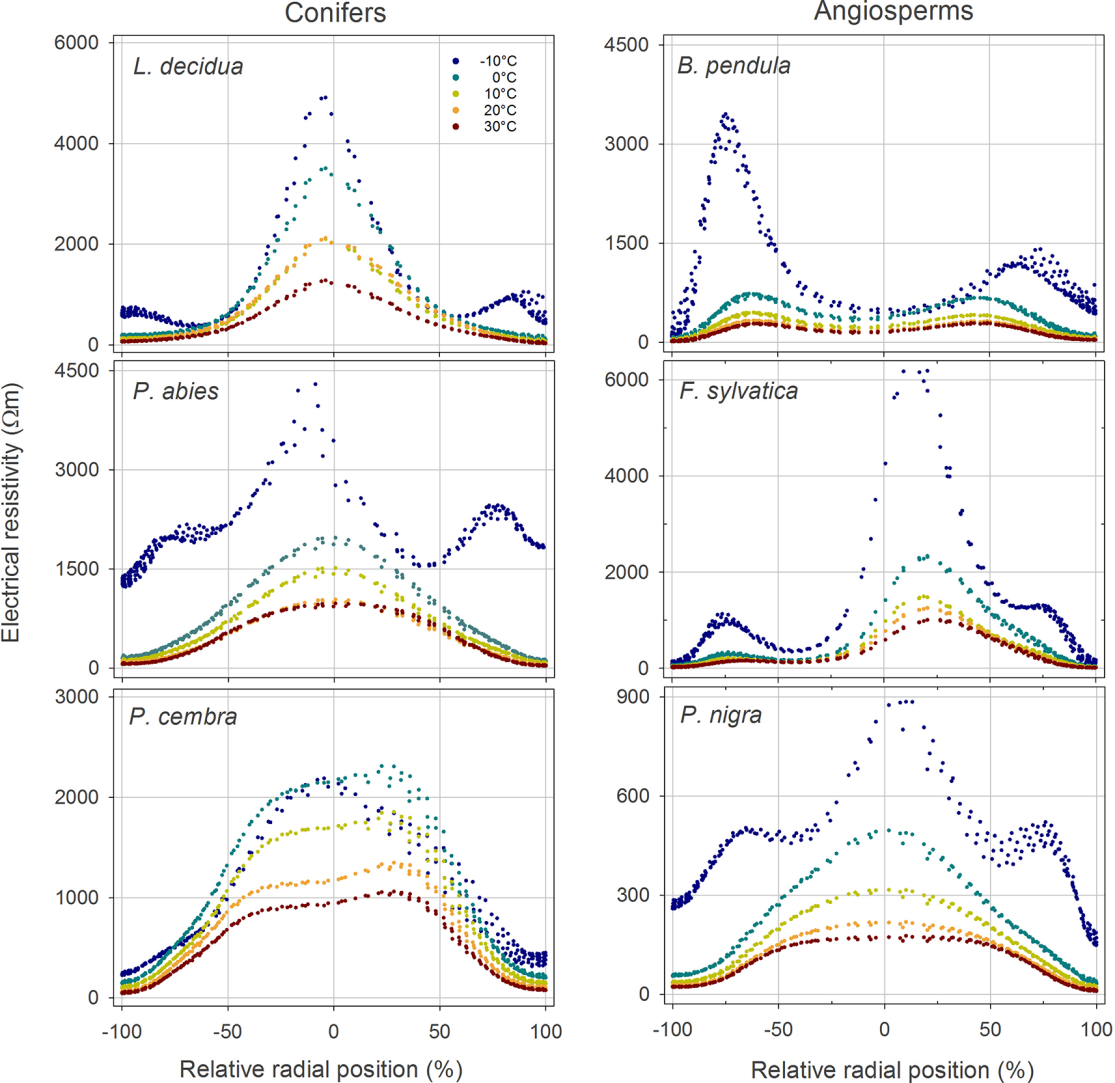
Radial distribution of the electrical resistivity (ER) in the stem cross section of analyzed tree species at different temperatures (see color legend). Values were extracted from tomograms shown in **Figure 1**. Absolute ER values are plotted against their relative position (0% = stem center, ± 100% = youngest annual growth ring) to account for differences in absolute stem diameter.

In all analyzed species, ER tomograms and profiles revealed lowest resistivities in the outer sapwood regions and a pronounced increase in ER toward the center (**Figures 1** and **2**). ER in conifer stems was overall higher than in angiosperms. While conifers exhibited a quite homogeneous and concentric pattern with a central ER peak, the distribution of ER was more variable across angiosperms and normally showed a slight decrease in ER in the stem center.

### Temperature Effects

In both angiosperms and conifers, measured resistivities increased with decreasing temperature (**Figure 1** and **Supplementary Table S1**). From 30 to 0°C, ER_mean_ increased 2.5-fold on average, following an exponential function which was similar in conifers and angiosperms (**Supplementary Figure S1**). Changing temperatures did not affect the general cross-sectional ER pattern and caused no distinct aberrations in tomograms, although increases were on average slightly higher in peripheral than in central areas. Passing the freezing point, a distinct, up to 14-fold (*P. abies*) raise in ER_mean_ was observed. This increase was mainly caused by a pronounced increase in ER_peripheral_, while the change in ER_center_ was comparably moderate (**Figure 2** and **Supplementary Figure S1**).

### Dehydration Effects

**Figure 3:**
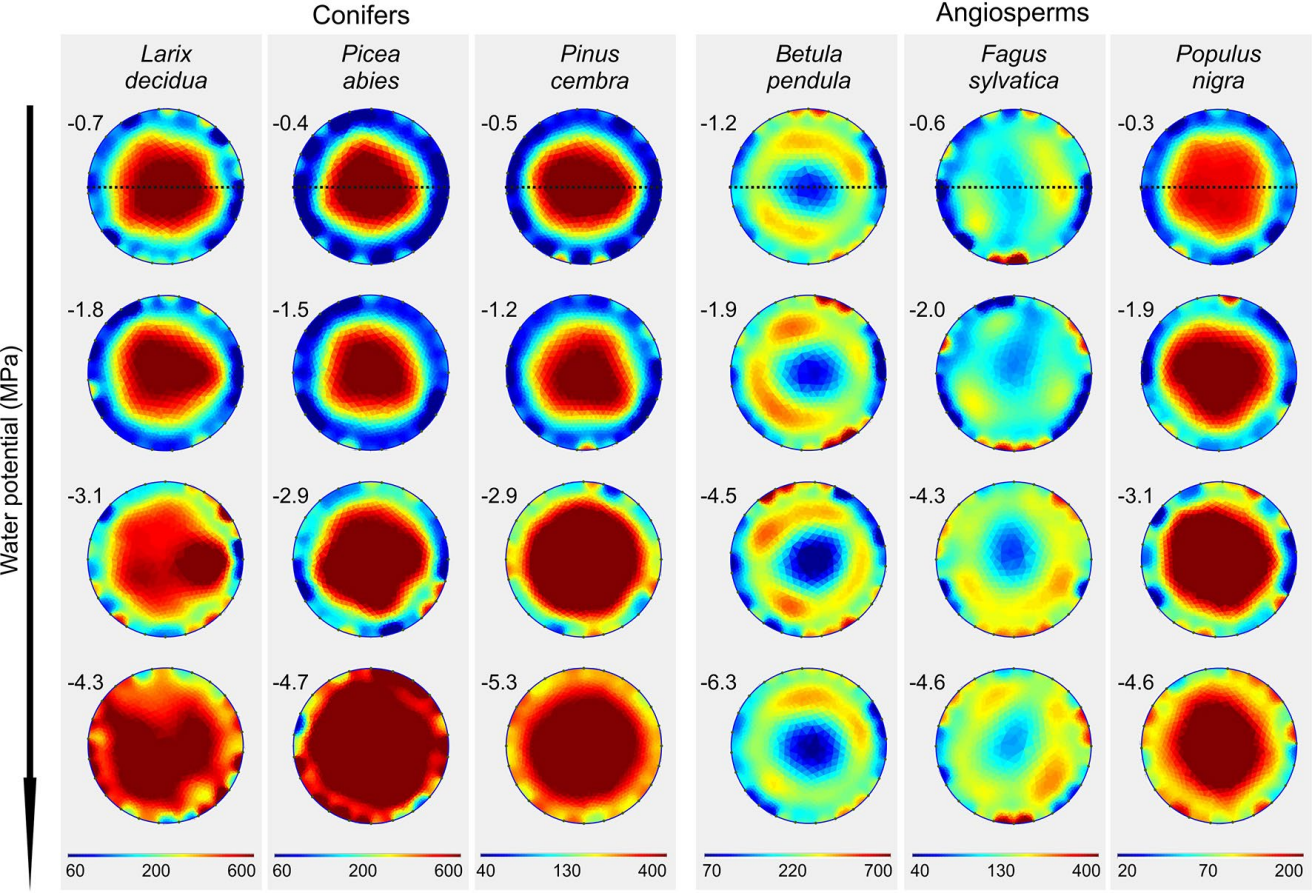
Electrical resistivity (ER) tomograms of the six analyzed species at varying water potentials (MPa; given on the left top of each tomogram). Blue colors indicate areas of low, and red of high ER (Ωm; note the species-specific range of the scale). Dashed lines indicate the orientation of radial ER profiles shown in **Figure 4**.

**Figure 4:**
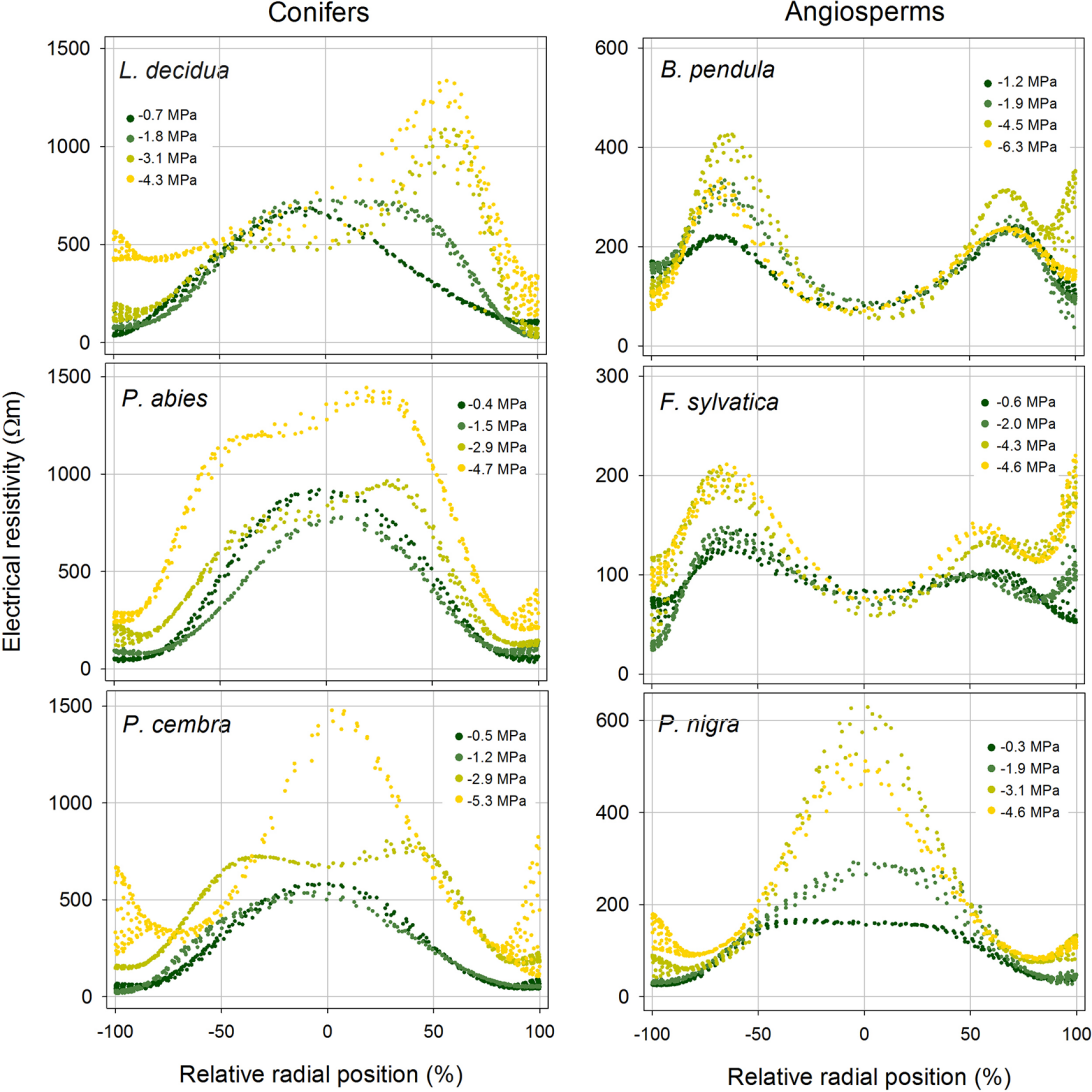
Radial distribution of the electrical resistivity (ER) in the stem cross section of analyzed tree species at different water potential (see color legend). Values were extracted from tomograms shown in **Figure 3**. Absolute ER values are plotted against their relative position (0% = stem center, ± 100% = youngest annual growth ring) to account for differences in absolute stem diameter.

With progressive dehydration, ER_mean_ increased in all study species, except *B. pendula*, which exhibited an overall constant ER_mean_ of approximately 200 Ωm (**Figure 3** and **Supplementary Table S2**). Conifers showed highest variation with an up to two-fold increase in *L. decidua* (at −4.3 MPa), 2.6-fold in *P. abies* (at −4.7 MPa), and 2.5-fold in *P. cembra* (at −5.3 MPa; **Supplementary Table S2**). Interestingly, the increase in resistivity occurred unevenly within the cross section in all species. This resulted in distinct alterations of ER patterns, also visible in ER profiles (**Figure 4**). Main changes were observed in the peripheral sapwood (all species) and in the stem center (all species except *B. pendula*, *F. sylvatica*). Furthermore, several distortions and irregular patches of high and low resistivity were observed in tomograms of dehydrated trees (**Figure 3**), especially when water potentials dropped below approximately −2 MPa.

**Figure 5:**
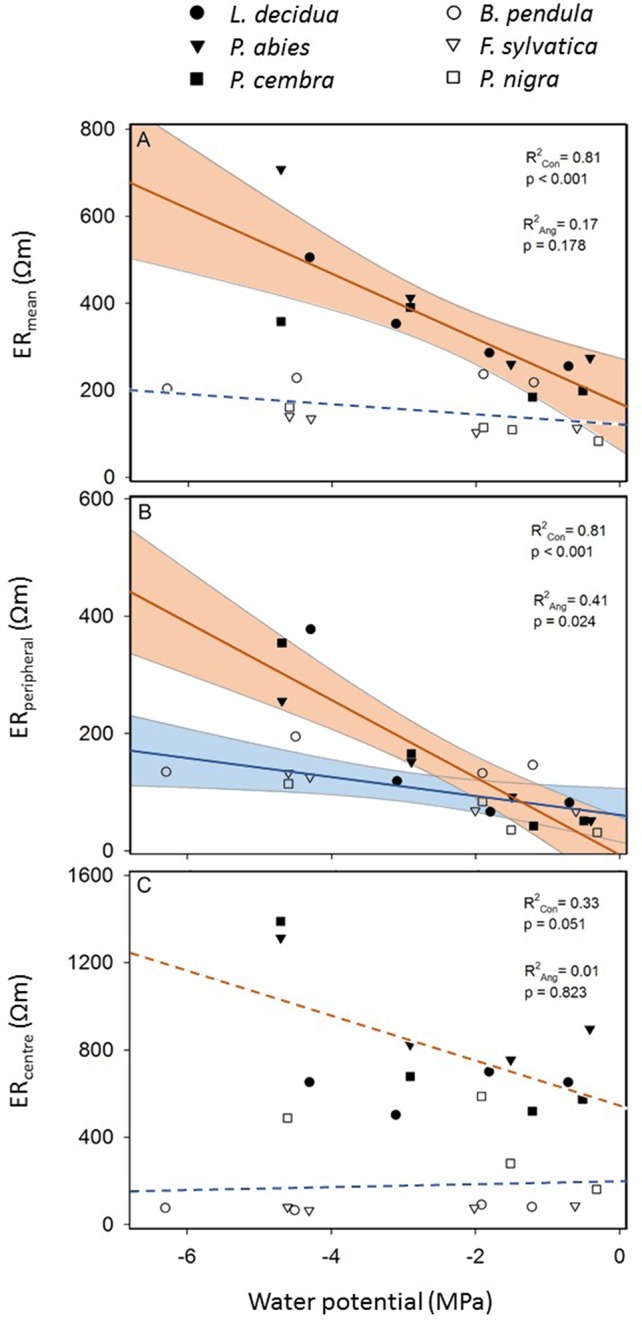
Linear regression of electrical resistivity (ER) versus water potential of conifers (filled symbols and red line; see species legend) and angiosperm trees (open symbols and blue line; see species legend). Shown are **(A)** the average ER of the entire cross section (ER_mean_) **(B)** the average ER of the peripheral ring between 90 and 95% relative radial position (ER_peripheral_), and **(C)** the average ER of the central area within 0–10% relative radial position (ER_center_). Solid lines indicate significant linear regressions (P ≤ 0.05) and shaded areas the 95% confidence interval.

Linear regression analysis (**Figure 5**) revealed a clear influence of tree water status on measured resistivities with more pronounced trends in conifers than in angiosperms. In conifers, ER_mean_ and ER_peripheral_ were highly dependent on the water potential; in angiosperms this relationship was statistically significant only for ER_peripheral_. In contrast, neither in conifers nor in angiosperms ER_center_ correlated with the water potential (**Figure 5**).

### Effects of Bark Removal and Electrode Installation

**Figure 6:**
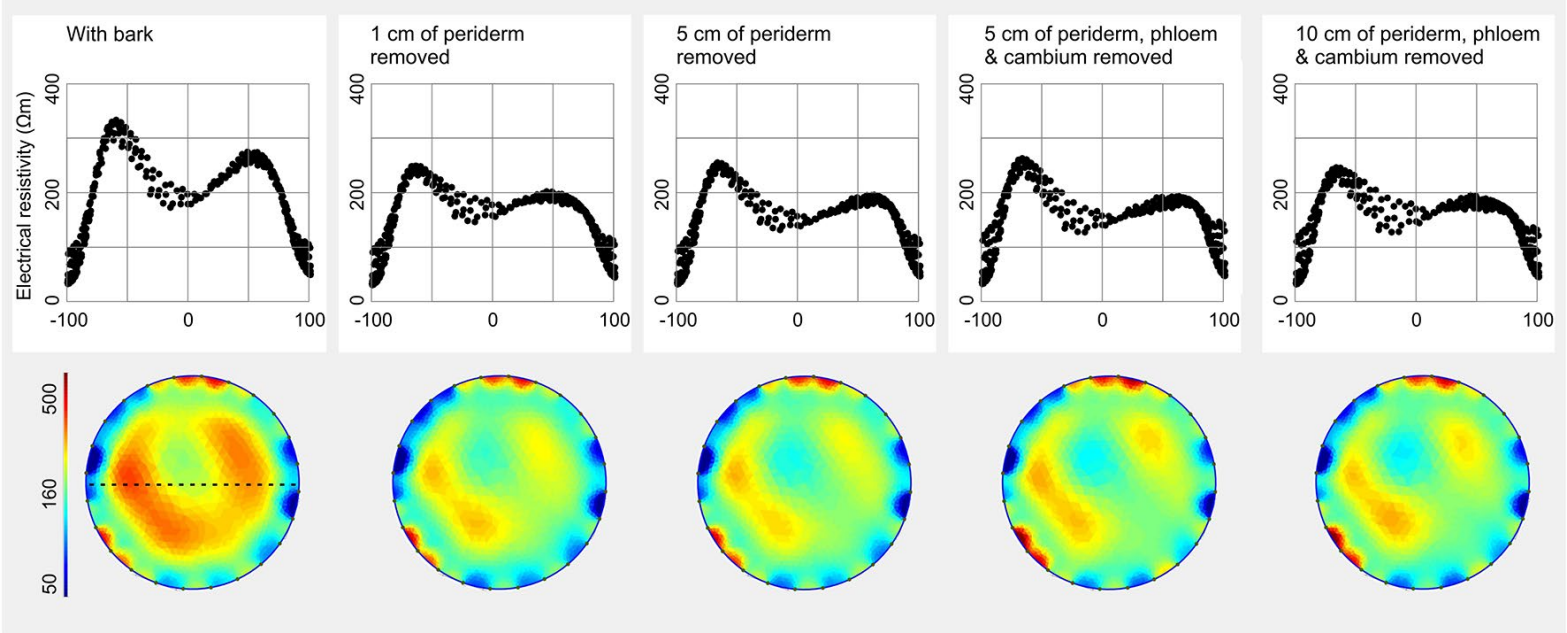
Influence of the bark on the electrical resistivity (ER) tomogram and profile of *Fagus sylvatica*. A ring of periderm, phloem, and cambium was removed to an increasing extent (1–10 cm above and below the installed electrodes, respectively). Blue colors indicate areas of low, and red of high ER (Ωm). For each tomogram, ER values were excerpted along a chosen diameter (orientation see dashed line in first tomogram) to generate the corresponding profile and were displayed in accordance to their relative position (0% = stem center, ± 100% = youngest annual growth ring).

Removal of the periderm resulted in a slight decrease of ER_mean_ in *F. sylvatica* (**Figure 6** and **Supplementary Table S3**). This effect could be observed already when 1 cm of the periderm and older phloem tissues was removed, and did not further change with progressive removal of larger bark areas, including also the phloem and cambium. Periderm removal affected mainly the high resistivity areas in the central part of the tomogram, while low ER of the outer peripheral ring remained constant.

**Figure 7:**
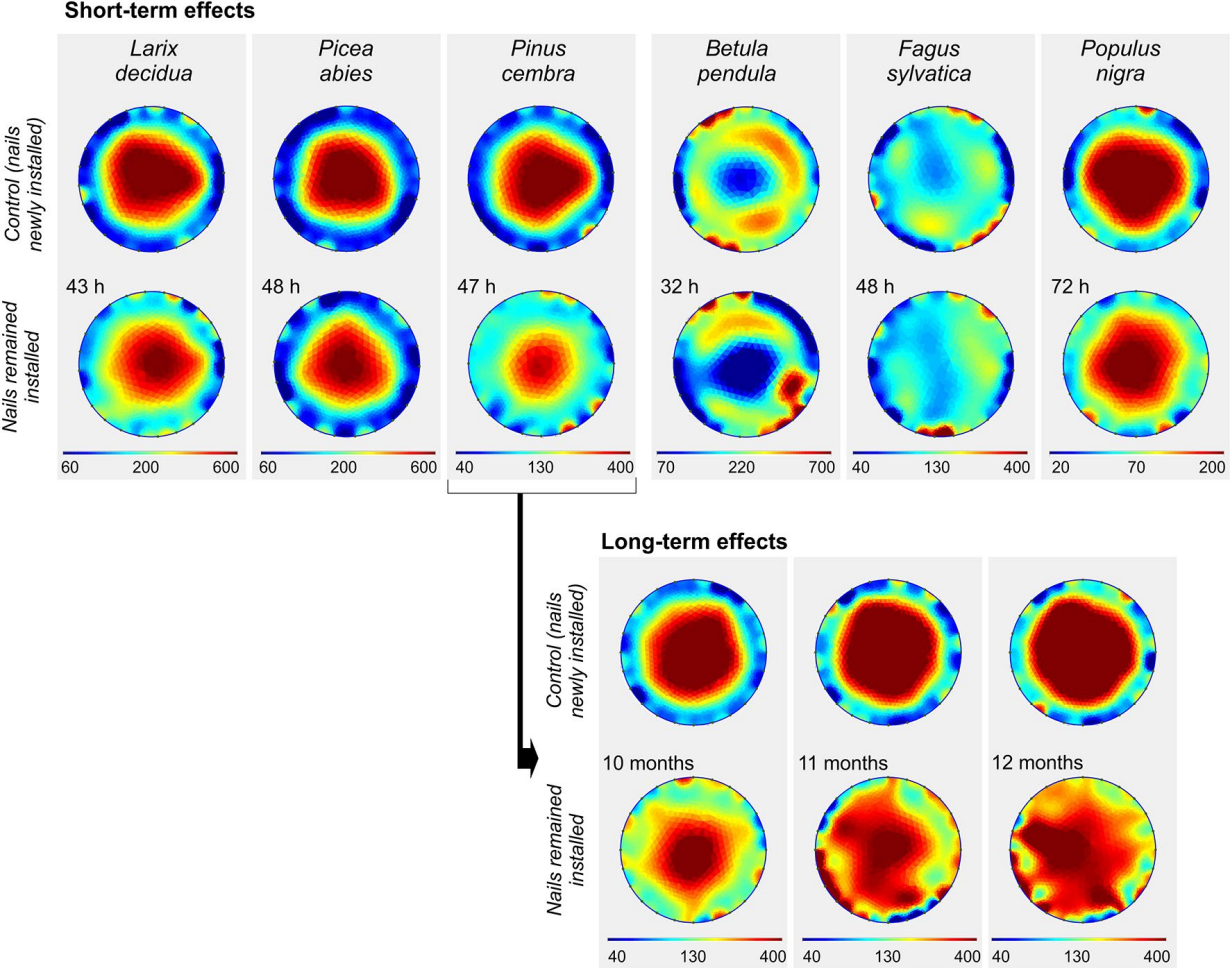
Effects of short- and long-term electrode installation on the electrical resistivity (ER) tomogram of analyzed tree species. For control measurements, new electrodes were installed directly before measurement a few centimeters below or above the old electrodes. Blue colors indicate areas of low, and red of high ER (Ωm; note the species-specific range of the scale).

Installation of electrodes for a short period of 32–72 h before conducting the tomography resulted in small changes of average ER (**Figure 7** and **Supplementary Figure S2**). The maximum effect was found in *P. cembra* with a decrease of 25% in ER_mean_ compared with the control measurement (**Supplementary Table S4**). A small decrease in ER was detected also for the other species, except for *F. sylvatica*, which showed a small increase. Although ER distribution patterns were overall similar to control measurements, small distortions and irregular patches of high and low resistivity in the peripheral ring were observed few days after electrode installation. However, changes in average ER and cross-sectional patterns were much more pronounced after a period of several months between electrode installation and ER measurements, as tested on a living tree of *P. cembra* in the field (**Figure 7**). ER distribution patterns showed serious distortions in all cross-sectional areas compared with the clear concentric ER gradient obtained *via* control measurements. After 10 and after 11 months, ER_mean_ was about 15% lower, while in September, after 12 months, ER_mean_ was 30% higher than measurements with new electrodes (**Supplementary Table S4**).

## Discussion

The present study demonstrated significant variation of ER levels and/or cross-sectional distribution patterns of tree stems with i) changing temperature and ii) continuing tree dehydration, and variable effects of iii) bark removal and electrode installation time. According to our hypothesis, temperature effects were most pronounced below the freezing point, but we also observed temperature-dependent changes in ER levels above 0°C. As expected, dehydration led to changes in ER levels and patterns, whereby some general differences between conifers and angiosperms were found.

(i) Temperature variation above 0°C did not affect the overall cross-sectional distribution pattern in all analyzed species (**Figures 1** and **2**), but ER_mean_ exponentially increased approximately 2.5-fold within the temperature range of 30–0°C in all species (**Supplementary Figure S1**). This general alteration of resistivity with temperature can be attributed to a reduced viscosity and increased ion mobility at higher temperatures (Tattar and Blanchard, 1976; Torgovnikov, 1993). The extent of this effect may depend on the water and ion content of the wood, as indicated by slightly higher increases in ER_peripheral_ (3-fold) than in ER_center_ (2.5-fold; **Supplementary Figure S1**). At temperatures below 0°C, free water (and partially also bound water) in the xylem will freeze. In ice, the mobility of electrons is strongly reduced, and thus its electric properties differ significantly from those of free water. This explains the dramatic changes in ER tomograms from 0 to −10°C (**Figure 1**), which were already described in previous studies (Larsson et al., 2004). Interestingly, the increase in ER_mean_ was accompanied in most species by an additional distinct increase in ER_peripheral_, while values of ER_center_ were almost in line with the exponential regression found above 0°C (**Figure 2** and **Supplementary Figure S1**). This phenomenon probably is based on the high water content of sapwood, which caused large changes in ER tomograms upon freezing. ER tomography of frozen stems thus might enable a more accurate determination of the sapwood area, because the transition pattern from high to low ER in the radial profile was more pronounced than at higher temperatures (**Figure 2**). We conclude that temperature dependence of ER measurements above the freezing point is of little relevance for tree assessment, because the detection of wood decay is mainly based on the occurrence of unexpected high and low ER patches and ER patterns remained overall unaffected by temperature. In contrast, consideration of temperature is critical with respect to absolute ER values and thus for physiological interpretations, like estimations of wood electrolyte or water content.

(ii) Tree tomography after drought periods were performed and/or critically discussed by several authors (e.g. Larsson et al., 2004; Guyot et al., 2013; Martin and Günther, 2013). Our study demonstrates that dehydration in fact causes considerable changes in ER_mean_ and ER patterns of tree stems (**Figures 3** and **4**). For tree assessment, we thus suggest to perform ER measurements on fully hydrated trees and, if possible, to conduct supplemental water potential measurements to exclude dehydration-induced ER bias. In all species (except *B. pendula*), dehydration caused an increase in ER_mean_, while changes in ER patterns differed largely across species. Water possesses anomalously high dielectric properties and thus has a large influence on the dielectric properties of wood, especially when portions of free, adhesive, and capillary water change (Torgovnikov, 1993). However, Tattar et al. (1972) pointed out that, as long as sufficient free water allows ion movement, ER of plant tissues is primarily affected by the concentration of mobile ions and relatively independent of small changes in tissue water content, while ER becomes dependent on the moisture content when free water is limiting. Asymmetric changes in the cross-sectional ER pattern thus may be attributed to this phenomenon or could indicate water shifts within the trunk due to inhomogeneous dehydration. It is also important to note that ER data are ambiguous, which means that more than one ER distribution pattern can explain the measured resistivities, although used algorithms help to keep errors small (Martin and Günther, 2013). The lack of an increase in stem ER with decreasing water potential in *B. pendula* is consistent with the results presented by Bär et al. (2019), who found no correlation between ER and wood moisture content for this species. As the same study also reported no relations between ER and electrolyte content or wood density, we can only speculate that there is an additional factor affecting measured ER in birch.

Despite all the aforementioned complicated interrelations between the wood water and ion content, a clear linear relationship between water potential and ER_mean_ was detected in conifers and angiosperms, respectively (**Figure 5**). Water potential was used in our study as a measure for dehydration for a variety of reasons: First, it is the relevant parameter in tree physiology representing water availability (Slatyer and Taylor, 1960), and directly related to the risk of hydraulic dysfunctions, such as cell turgor loss and xylem cavitation. Second, up to now it has never been tested if the ER technology may be used to estimate tree water potentials, while the linkage between ER and wood moisture content was well-known (Bieker and Rust, 2010b; Guyot et al., 2013; Bär et al., 2019). Since drought is a major stress factor for trees worldwide and expected to increase due to climate change, nondestructive methods for tree water status would be highly desirable. The statistical analysis revealed that the relationship is more pronounced in conifers than in angiosperm species and more clearly in peripheral stem areas (i.e. sapwood) than in the center (**Figure 5**). Although accurate quantification of water deficits by ER tomography will remain challenging, a potential application for conifers, with the focus on sapwood areas, seems promising. Most significant changes in tomograms were detected between −2 and −4 MPa (**Figure 3**), which is in the range of water potentials inducing embolism in the analyzed species. The water potential causing 50% loss of conductivity is approximately −2.3 MPa in *B. pendula* and between −3.2 and −3.6 MPa in the other species (Choat et al., 2012), and represents an important threshold for the estimation and prediction of drought-induced tree damages. Observed changes in ER around these water potentials thus might be directly related to embolism formation and respective air-filled conduits in the xylem. Like in hydrogeophysical techniques, which monitor the dynamic behavior of soil water by repeated measurements during infiltration or growth experiments (Al Hagrey, 2007), ER tomography might be useful to noninvasively record the most critical dehydration periods and resulting development of xylem dysfunction in conifer tree stems. Though, additional experiments and field tests are required to set up a reliable methodical approach.

(iii) ER tomography visualizes resistivities in the trunk xylem, whereby influences of tissues outside the xylem are expected to be negligible. However, because electrons do not follow necessarily the shortest way between two electrodes but may pass through the bark, a potential effect cannot be excluded (Nicolotti et al., 2003). This aspect is complicated by the fact that bark is an even more heterogeneous material than wood, consisting of several layers with contrasting moisture content and species- as well as age-specific thickness (Rowell, 2013). Furthermore, cambium and phloem contain a lot of water and many ions (Torgovnikov, 1993) and may thus exhibit high electric conductivity. As the activity of these tissues can considerably change with season and life time, their influence on ER seems especially critical. Our measurements on *F. sylvatica* revealed slightly higher maximum ER peaks in the stem with intact bark than in the decorticated stem (**Figure 6**). In contrast, presence or absence of phloem and cambium did not affect the measurement. Results indicate that high resistivities in the periderm slightly affect the electron transport and provoke higher measured ER values but no aberrations in the cross-sectional ER pattern. It remains to be clarified for species with tertiary bark tissue, if thicker peripheral bark layers have stronger impacts on ER tomography measurements.

Small peripheral arcs with high ER, usually situated on opposite stem parts, probably were related to reaction wood (compression wood in conifers and tension wood in angiosperms; Bieker and Rust, 2010a), which is characterized by higher wood density and diverging lignin or cellulose content. Local ER variations near the electrode positions were probably caused by the lower density of measurement lines in the periphery and by higher electron transport resistances in the tangential than the radial direction.

Physiological analyses frequently require repeated measurement of the same tree over a certain period of time. Results of the present study indicate that tomograms are affected if electrodes are not inserted directly before measurements but remain installed in the stem for several days or months (**Figure 7**). Short-term installation of a few days (under laboratory conditions) only slightly affected the tomograms, while long-term installation over several months (in the field) caused small changes in ER_mean_ but obvious alterations in the tomogram pattern of *P. cembra*. Interestingly, ER_mean_ showed only relative small changes during the first 11 months until August (see **Figure 7**). In September (after 12 months) ER_mean_ increased substantially compared to control measurements, potentially due to lower temperatures and the onset of stem freezing in this period. Although the wood injury caused by inserting nails is considered marginal, small damages can cause wood defense reactions like tyloses formation, deposition of suberin, pectin, and phenolic compounds, and lignification of surrounding cells (Rioux et al., 1998; Maranon-Jimenez et al., 2018). Such reactions are known to be highly species-specific, and can be accompanied, especially in conifers, by secretion of resin (Stoffel and Klinkmüller, 2013). Another possible explanation for local increases in ER is the entering of air and subsequent embolization of xylem conduits (Sperry and Tyree, 1988). Also polarization effects (accumulation of ions around the electrodes; Tattar and Blanchard, 1976) can alter the electrical properties of the tissue around, although this effect is minimized by using electrodes of inert metals. We conclude that effects of short-time installation of electrodes will not be relevant for tree assessment but critical for detailed physiological analyses, while long-term installation should be avoided whenever possible.

## Conclusions

The present study confirms that ER tomography of tree stems is sensitive to temperature and water status, and thus both factors have to be considered for accurate interpretation of measurements. However, since temperature and dehydration dependence of ER tomograms can be quantified, variations potentially can be corrected or even evaluated to extract useful information with regard to those parameters. Results indicate that the method may be suitable to accomplish nondestructive determination of the hydraulic status of tree stems and can complement ER measurement of soil and root networks. Thus, ER tomography has a large potential for presymptomatic detection of physiological stress in standing trees and represents an appealing data source for both scientists and forest managers. The present study underlines that it may be further developed to provide an efficient and reliable measure of tree responses to changing climatic conditions.

## Data Availability Statement

All datasets generated for this study are included in the article/**Supplementary Material**.

## Author Contributions

Conceived and designed the experiments, contributed to the writing and revision of the manuscript: SM, AG, JS, AB, and AL. Performed the experiments and analyzed the results: JS and AG.

## Funding

This study was supported by the Austrian Science Fund (FWF) P29896-B22.

## Conflict of Interest

The authors declare that the research was conducted in the absence of any commercial or financial relationships that could be construed as a potential conflict of interest.

## Abbreviations

ER, electrical resistivity; ER_mean_, average ER of the entire cross section; ER_peripheral_, average ER of the peripheral ring between 90 and 95% relative radial position; ER_center_, average ER of the central area within 0–10% relative radial position.
