# Supplementary material for: Noninvasive Analysis of Tree Stems by Electrical Resistivity Tomography: Unraveling the Effects of Temperature, Water Status, and Electrode Installation
Source: Front Plant Sci. 2019 Nov 13;10:1455. doi: 10.3389/fpls.2019.01455 (PMC6865845; doi:10.3389/fpls.2019.01455)
Supplement: Supplementary file 1 [file DataSheet_1.pdf]

Supplementary Material

Noninvasive Analysis of Tree Stems by Electrical Resistivity Tomography: Unravelling the Effects of Temperature, Water Status, and Electrode Installation

Andrea Ganthaler, Julia Sailer, Andreas Bär, Adriano Losso, Stefan Mayr

Supplementary Table S1. Electrical resistivity (ER) parameters (mean ± SE, minimum and maximum) for tomograms performed at different temperatures (Temp.). For each tree the stem diameter (Diam.) at measurement height is given.

Tree species	Diam. (mm)	Temp. (°C)	ER_{mean} (Ωm)	ER_{max}(Ωm)	ER_{min} (Ωm)
B. pendula	56.3	**30**	136.34 ± 1.36	755.33	8.66
		**20**	158.17 ± 1.53	891.58	10.22
		**10**	211.00 ± 2.00	1057.88	14.59
		**0**	336.94 ± 3.39	1697.66	21.87
		**-10**	1271.67 ± 32.70	28803.40	85.38
F. sylvatica	46.8	**30**	265.08 ± 4.60	1007.48	8.07
		**20**	316.35 ± 5.63	1380.61	10.00
		**10**	394.08 ± 6.86	2610.23	15.85
		**0**	613.39 ± 10.82	4765.38	24.85
		**-10**	2174.14 ± 75.88	61033.30	55.80
P. nigra	63.7	**30**	80.22 ± 0.94	177.82	8.97
		**20**	92.95 ± 1.09	218.31	11.78
		**10**	124.82 ± 1.50	315.64	18.68
		**0**	179.73 ± 2.11	494.95	30.87
		**-10**	454.96 ± 2.98	2094.30	147.86
L. decidua	55.1	**30**	276.53 ± 5.18	1279.42	22.04
		**20**	421.05 ± 8.66	2123.36	27.73
		**10**	423.01 ± 8.13	2099.79	41.83
		**0**	628.61 ± 13.07	3506.59	77.24
		**-10**	930.19 ± 15.14	6505.26	209.52
P. abies	55.1	**30**	314.89 ± 5.19	975.58	25.65
		**20**	327.47 ± 5.01	1033.78	36.63
		**10**	447.94 ± 6.85	1510.28	61.87
		**0**	607.61 ± 8.90	1969.88	90.45
		**-10**	4455.07 ± 26.82	9216.53	1501.87

P. cembra	43.0	**30**	119.74 ± 1.54	350.47	27.25
		**20**	122.05 ± 1.20	252.17	44.13
		**10**	206.44 ± 2.72	636.31	56.54
		**0**	330.38 ± 3.82	896.14	80.52
		**-10**	855.02 ± 9.59	2378.29	94.00

Supplementary Table S2. Electrical resistivity (ER) parameters (mean ± SE, minimum and maximum) for tomograms performed at varying tree water potential (Ψ). For each tree the stem diameter (Diam.) at measurement height is given.

**Tree species**	**Diam. (mm)**	**Ψ (MPa)**	**ER_{mean} (Ωm)**	**ER_{max}(Ωm)**	**ER_{min} (Ωm)**
B. pendula	52.5	**-1.2**	201.93 ± 1.44	743.86	35.63
		**-1.9**	236.71 ± 2.03	1386.00	21.48
		**-4.5**	227.90 ± 1.90	1089.82	34.21
		**-6.3**	203.52 ± 1.26	390.85	63.94
F. sylvatica	49.3	**-0.6**	112.66 ± 1.61	1913.87	8.20
		**-2.0**	103.16 ± 0.71	402.53	14.11
		**-4.3**	134.54 ± 0.69	318.06	33.32
		**-4.6**	140.01 ± 0.82	569.87	44.82
P. nigra	42.3	**-0.3**	83.10 ± 0.98	167.10	11.40
		**-1.9**	108.93 ± 1.58	291.69	8.55
		**-3.1**	114.10 ± 1.56	267.56	11.81
		**-4.6**	160.33 ± 2.20	523.45	30.81
L. decidua	52.5	**-0.7**	253.58 ± 2.95	685.63	34.37
		**-1.8**	284.35 ± 3.88	726.63	26.25
		**-3.1**	351.16 ± 3.53	1121.38	19.64
		**-4.3**	503.66 ± 3.80	1335.02	99.25
P. abies	43.0	**-0.4**	272.47 ± 5.08	918.55	14.37
		**-1.5**	258.35 ± 4.26	778.21	14.69
		**-2.9**	411.05 ± 5.65	976.06	31.55
		**-4.7**	706.40 ± 8.91	1655.50	66.74
P. cembra	47.7	**-0.5**	196.70 ± 3.19	581.84	12.98
		**-1.2**	182.76 ± 2.65	539.68	15.98
		**-2.9**	388.56 ± 4.58	846.30	47.94
		**-5.3**	484.78 ± 5.86	1689.41	47.84

Supplementary Table S3. Electrical resistivity (ER) parameters (mean ± SE, minimum and maximum) for tomograms performed following progressive bark removal on a *Fagus sylvatica* stem.

Tree species	Diam. (mm)	Bark removal	ER_{mean} (Ωm)	ER_{max}(Ωm)	ER_{min} (Ωm)
F. sylvatica	76.0	with bark	194.18 ± 1.09	396.04	24.13
		1 cm periderm	160.87 ± 0.82	445.22	21.79
		5 cm periderm	161.12 ± 0.80	436.92	23.41
		5 cm periderm, phloem & cambium	168.29 ± 0.92	607.40	24.14
		10 cm periderm, phloem & cambium	162.96 ± 0.89	585.11	23.30

Supplementary Table S4. Electrical resistivity (ER) parameters (mean ± SE, minimum and maximum) for tomograms of trees performed immediately (control) or with delay (32 – 72 hours) after electrode installation. Additional measurements on *P. cembra* after 10 – 12 months were obtained on a tree in the field. For each tree the stem diameter (Diam.) at measurement height is given.

Tree species	Diam. (mm)	electrode inst.	ER_{mean} (Ωm)	ER_{max}(Ωm)	ER_{min} (Ωm)
B. pendula	56.3	control	236.71 ± 2.03	1386.00	21.48
		32h	215.90 ± 1.44	743.86	35.63
F. sylvatica	46.8	control	103.16 ± 0.71	402.53	14.11
		48h	107.20 ± 1.90	2310.61	11.69
P. nigra	63.7	control	108.93 ± 1.58	291.69	8.55
		72h	96.49 ± 1.03	206.51	16.99
L. decidua	55.1	control	284.35 ± 3.88	726.63	26.25
		43h	255.53 ± 2.70	647.66	36.15
P. abies	55.1	control	258.35 ± 4.26	778.21	14.69
		48h	228.23 ± 3.28	645.33	19.67
P. cembra	43.0	control	182.76 ± 2.65	539.68	15.98
		47h	137.89 ± 1.26	355.22	38.26
P. cembra	70.0	control	232.52 ± 3.34	660.38	23.88
		10 months	202.07 ± 1.86	515.49	55.91
		control	300.63 ± 4.98	1028.81	32.09
		11 months	253.26 ± 2.11	523.72	26.49
		control	383.39 ± 6.69	1354.31	22.58
		12 months	502.20 ± 8.35	2184.46	17.41

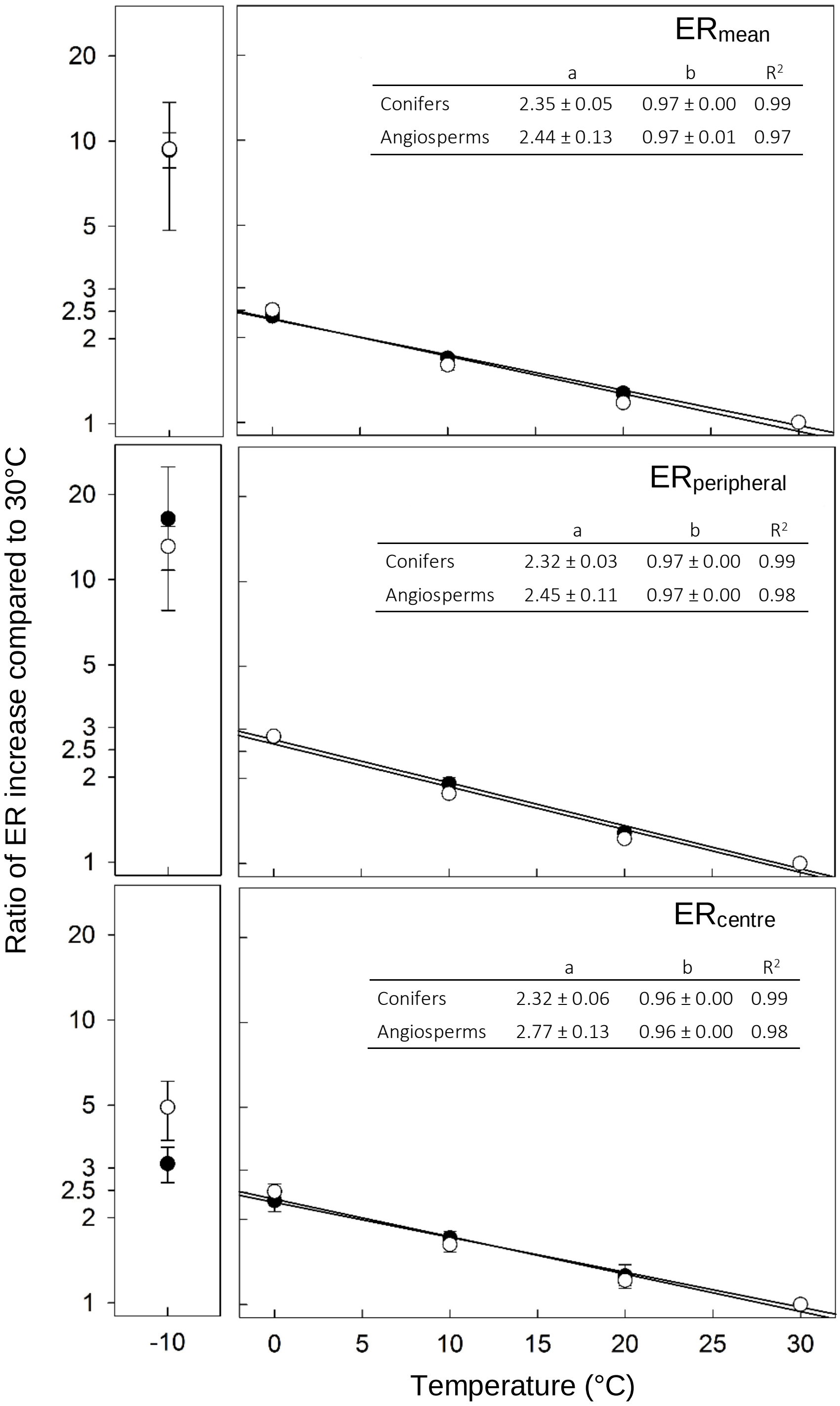

Supplementary Figure S1. Temperature induced changes in electrical resistivity (ER) for conifers (filled symbols) and angiosperms (open symbols) in the entire cross section (ER_{mean}), in the peripheral ring between 90-95% relative radial position ($ER_{peripheral}$), and in the central area within 0-10% relative radial position (ER_{center}). Ratio of ER increase was calculated by dividing ER measured at -10, 0, 10 and 20°C, respectively, by ER measured at 30°C for each species and by calculating mean values and standard errors for conifers and angiosperms. Solid lines indicate exponential regression lines ($f(x) = a^* b^x$) and respective parameters are given in each panel. Not that the y-axis (ratio of ER changes) is plotted on a log scale.

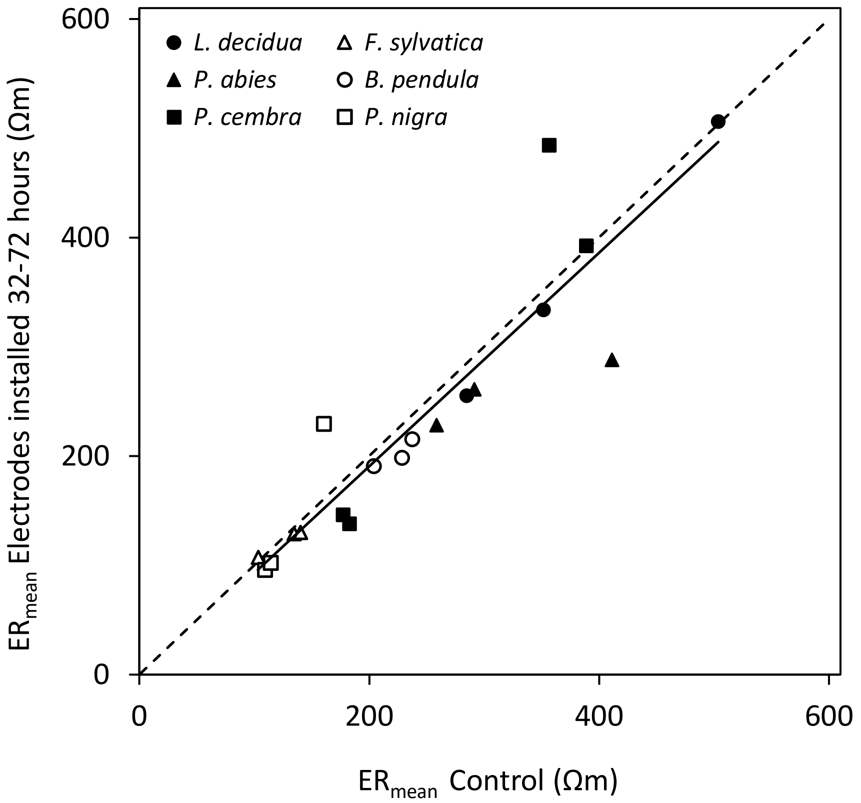

Supplementary Figure S2. Effects of short-term electrode installation on mean cross-sectional electrical resistivity (ER_{mean}). Values obtained using newly installed electrodes (Control) were compared with measurements obtained on electrodes installed for 32 – 72 hours. Presented data include measurements shown in Fig. 6 and two additional measurements per species, respectively. The dashed line indicates the concordance line and the solid line the linear regression between the two ER measurements (R^2 = 0.85).
